# A school-based physical activity program to improve health and fitness in children aged 6–13 years ("Kinder-Sportstudie KISS"): study design of a randomized controlled trial [ISRCTN15360785]

**DOI:** 10.1186/1471-2458-6-147

**Published:** 2006-06-06

**Authors:** Lukas Zahner, Jardena J Puder, Ralf Roth, Marco Schmid, Regula Guldimann, Uwe Pühse, Martin Knöpfli, Charlotte Braun-Fahrländer, Bernard Marti, Susi Kriemler

**Affiliations:** 1 Institute of Exercise and Health Sciences, University of Basel, Brüglingen 33, 4052 Basel, Switzerland; 2 Division of Endocrinology, Diabetes & Clinical Nutrition, University Hospital of Basel, 4031 Basel, Switzerland; 3 Institute for Social and Preventive Medicine, University of Basel, Steinengraben 49, 4051 Basel, Switzerland; 4 Swiss Federal Office of Sports, 2532 Magglingen, Switzerland

## Abstract

**Background:**

Childhood obesity is the result of a long lasting imbalance between energy intake and energy expenditure. A major contributing factor is physical inactivity which is closely linked to bone health, cardiovascular disease risk, fitness and psychological factors. The school seems to provide an excellent setting to enhance levels of physical activity (PA). However, there is insufficient data from previous school-based intervention trials on how to enhance overall PA. It is also unknown whether an intervention aimed at increasing PA is effective in improving the children's health. The purpose of this paper is to outline the design of a school-based randomized, controlled trial (RCT) aiming to increase overall PA and to improve fitness and health in 6- to 13-year-old children.

**Methods/Design:**

15 schools were randomized to the intervention (n = 9) or the control (n = 6) group, stratified by geographic region (urban vs. rural) and by age (1^st ^and 5^th ^grade). Participation was given for all children in the intervention group since in this group the intervention was part of the normal school curriculum. The intervention during one academic year consisted of: 1. two additional physical education classes per week given by trained physical education teachers adding up to a total of five PA classes per week, 2. short PA breaks (2–5 min each) during academic lessons, 3. PA home work, and 4. adaptation of recreational areas around the school. All children underwent anthropometric measurements, blood pressure assessment, fitness testing, measurement of PA and they filled out questionnaires. At least 70% of all children agreed to blood sampling and measurements of body composition and bone mineral measurements by dual energy x-ray absorptiometry. The primary endpoints of the study after one year were an increase in total PA by accelerometry, an increase in aerobic fitness measured by the 20 m shuttle run, a decrease in percent body fat derived from skinfold measurements and an increase in quality of life as assessed by the child health questionnaire in the intervention group compared to the control group. Secondary outcomes were overall fitness, differences in body composition including body fat distribution, cardiovascular risk factors, psychosocial health, bone mineral content and density of femur, lumbar spine and total body and food intake.

**Discussion:**

Our preliminary data suggest that the children were representative of Swiss children with respect to sex, socio-demographic status, and body mass index. Short-term results can be expected by the beginning of 2007. We hypothesized that our intervention will lead to an increase in PA, fitness and overall health. Based on our data, we aim to provide important information regarding the influence of such an intervention on these outcome measures in school-aged children and to provide nationwide guidelines to improve PA in children.

## Background

### Initiation and realization of the research project

The debate regarding the ideal amount of physical education lessons that the state is ready to support is a major controversial issue in many countries, including Switzerland [1]. The importance of the growing problem of an inactive lifestyle in children, with the burden major health implications like obesity, type 2 diabetes, hypertension, low fitness and osteoporosis, is highly undervalued. The Swiss Federal Office of Sports (FOSPO) therefore decided to support an evaluation of the current level of physical activity (PA), fitness and health of elementary school children in Switzerland in addition to a school-based PA intervention program over the period of one academic year with a follow up three years later.

### Arguments for publishing a design paper

In this article we describe the design of a randomized controlled trial (RCT) assessing a school-based PA enhancing program which took place over the period of one school year. Publishing the design and rational of a RCT before the results are available has important benefits. The study can be critically evaluated for its methodological quality, irrespective of the results. If a design paper has been written, the results will most probably be published, even if the results are negative. A design paper includes a more detailed description of the study population, the intervention and all outcome measures than what can be reported in the method section of a regular publication focusing only on part of the study results. This paper will help researchers in the same field to compare the methods and the methodological qualities of future RCT and it provides detailed information for health professionals or educators to apply the intervention if the results show improvements in the outcome measures.

### Introduction

The increase in sedentary behavior, i.e. physical inactivity, over the last decades is thought to be one of the main risk factors for the development of obesity, diabetes, cardiovascular disease, osteoporosis and psychosocial constraints [2]. Despite widespread attempts to increase PA in the general population, only a minority of adults and children in developed countries engage in PA to a degree sufficient to maintain or increase health and physical as well as psychosocial well-being [3]. There is evidence that an insufficient amount of PA starts in childhood and tracks into adult life [4]. Risk factors which enhance sedentary behavior are large amounts of time spent in front of the television and/or computer, the inability to play outside or to actively commute to school, inactive parents or parents who do not support children to be active, and the lack of sufficient physical education at school. Although in children aerobic fitness is only moderately associated with PA, low PA and fitness are both associated with increasing prevalence of cardiovascular risk factors [5], even after adjusting for weight and/or obesity [5].

Currently, about 40–50% of the European adult population is overweight. Being overweight is an important risk factor for cardiovascular diseases, type 2 diabetes mellitus, musculo-sceletal disorders, and impaired quality of life [6]. The current epidemic of overweight and obesity is largely caused by an environment that promotes excessive food intake and discourages physical activity [7]. Even among children and adolescents, an alarming increase in overweight has been noticed. In 2002, the prevalence of overweight in 6- to 12-year-old Swiss children was 16.6 and 19.9% in boys and girls, respectively [8]. Even if obese adolescents become normal weight adults, they keep their burden of an increased cardiovascular risk [9]. Furthermore, there is a close link between (central) obesity and other cardiovascular risk factors like insulin resistance, hypertension, dyslipidemia and low-grade chronic inflammation in children and adolescents [5,10]. The prevalence of the metabolic syndrome has dramatically increased, and 46% of US adolescents have at least one risk factor of the metabolic syndrome [11]. Overweight children are also at increased risk to develop orthopedic complications and to suffer from stigmatization and discrimination [12].

PA does not only positively influence physiological factors, but has also a positive effect on psychological aspects. Fifty percent or even more of elementary school children report to be stressed or exhausted, and not able to sleep well [13]. Regular PA can increase the ability to cope with stress and leads to an improved health perception and quality of life [14]. In addition, a positive influence of PA on measures of anxiety and depressive symptoms [15] has been shown. PA does not only act on an individual level, but influences the school climate positively by increasing social competence within classes. This leads to an improved social behavior and more satisfaction with school, rated both by pupils and teachers [16]. Furthermore, regular PA can reduce substance abuse such as smoking [17]. Overweight children are particularly at increased risk to have a low self-esteem and to suffer from stigmatization and discrimination [12]. One report showed that the addition of regular PA lead to a higher self-esteem one year later in those children [18].

Maximizing bone mass during growth may constitute one of the most effective prevention strategies for osteoporosis, a disease which affects millions of people throughout the world. There is evidence that the degree of weight bearing activity and calcium intake is related to bone mass in childhood and adolescence [19,20]. Therefore, the benefits from PA in childhood may continue even into late adulthood [21].

Many school-based intervention studies promoting PA and a healthy lifestyle have been performed over the last two decades [22]. However, only a few studies documented increased PA during school-time [23,24]. In one study, even a reduction in out-of school PA as result of a PA program at school was observed [25]. In general, the results are disappointing, possibly due to methodological limitations, such as non-randomized trials, lack of objective measurements of PA and fitness, promotion of PA by non-experts and incomplete evaluation of cardiovascular risk factors [22,26]. In addition, a lack of overall increase in PA observed in many school-based intervention studies could be either due to compensation by increasing sedentary activity during out-of school time, or possibly due to the promotion of interventions that may not have been vigorous or sustained enough. A major advantage of interventions focusing on elementary school children is the relatively easy access through the schools, where changes in the school environment that promote healthy behaviors can be easier implemented and monitored, and can reach many schoolchildren of all social classes. Furthermore, the school environment gives the opportunity to work on various areas before, during and after school to increase overall and/or total PA.

## Methods/Design

### Study objectives

The aim of the proposed project was to evaluate in a RCT a school-based program if implementation of an extended PA program throughout the day during one school-year improved overall PA, physical fitness, physical health, and psychosocial well-being in 6- to 13-year-old children.

### Main outcomes

Primary outcomes of the intervention study were defined as

1. an increase in total PA measured by accelerometers,

2. an increase in aerobic fitness measured by the 20 m shuttle run test,

3. a reduction of percent body fat measured by skinfolds

4. an increase in quality of life measured by the child health questionnaire

in the intervention group compared to the control group.

Secondary outcomes of the intervention study were overall fitness (i.e. strength, speed, endurance, flexibility and coordination), body composition (including body fat distribution), cardiovascular risk factors (including blood pressure, blood lipids, glucose metabolism, C-reactive protein, adiponectin), bone health (including bone mineral content and density of femur, lumbar spine and total body as well as bone metabolism), psychosocial health (including social and stress coping, social anxiety, self esteem, drug abuse), and food intake. These outcomes were tested in different subgroups, i.e. 1^st ^vs. 5^th ^grade, girls vs. boys, children of different weight status, pubertal stages and socio-economic background.

### Study groups/Recruitment

**Figure 1:**
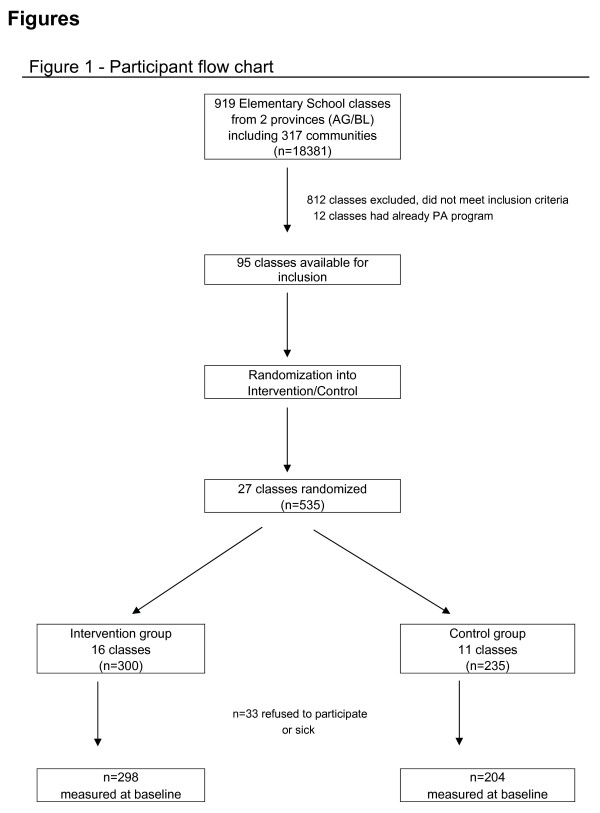
Participant flow chart.

Figure 1 shows the flow diagram of the recruited population. The study was performed in two provinces of Switzerland. Within these provinces, there were 919 elementary schools. Of these 95 fulfilled our stratification criteria, i.e. rural versus urban localization, and a prevalence of 10–30% children from other ethnicities. These strata were chosen in order to be representative for the Swiss population. Recruitment of participating schools (i.e. cluster randomization) was based on the willingness of these 95 elementary schools to be randomized either to an intervention group with a PA curriculum or a control group. Fifty percent of all children were in 1^st ^grade (6–8 years), and the other children were in 5^th ^grade (10–12 years) with evenly distributed gender. Fifteen schools with a total of 27 classes were randomized by a random-number table. Sixteen classes in 9 schools located in 6 communities were randomized to the intervention group (INT), and eleven classes in 6 schools in other communities of the same provinces were randomized to the control group (CON). A higher number of schools in the INT than in the CON group, i.e. a randomization ratio of 3:2 was chosen to gain more experience with the intervention and to reduce costs of the trial. Data of the INT and CON group were collected solely in the schools, in order to minimize the absence time from the usual school curriculum due to travelling.

Although the intervention program itself was established as part of the school curriculum for all children in the INT group, informed consent for the questionnaires and all measurements was necessary for all participants and was given by the children and a parent. The study was approved by the Ethics Committees of the University of Basel, the University of Zürich, as well as by the cantonal ethical committee of Aargau, Switzerland.

### Data analysis

To compare the success of randomization, descriptive statistics were used to compare the baseline measurements of the two groups. The primary analysis was a comparison of the change of the primary and secondary outcome factors between the intervention and the control group following the "intention to treat" principle. Initial power analysis was based on a two-sided t-test on the pre-post changes in the primary outcome measures (total PA, aerobic fitness, percent body fat, quality of life), with adjustments to account for the intraclass correlation (ICC) due to cluster randomization by school. The study was powered to detect a medium effect size on all primary study outcome measures of 0.5 units of standard deviation with 79% power if the ICC was 0.10 and with 90% power if the ICC was 0.06. Sample size required to reach the estimated power was a total of 360 children at the third measurement, four years after baseline assessment. Assuming an attrition rate of 10–15% and accounting for the multiple secondary outcomes, we recruited 530 children in the intervention and control group to provide adequate power to test/challenge the following null-hypothesis of the study: At the end of the intervention there will be no difference in the primary outcomes between the intervention and the control groups. Pre-planned subgroup analyses will be performed for age groups (1^st ^vs. 5^th ^grade), gender, weight status, pubertal stage, and socio-economic background. Where appropriate, adjustments were made for differences in baseline values. The same differences in the outcomes will be also tested 3 years later.

### Study organization

All INT groups proceeded according to the same curriculum, prepared by the expert physical education teachers. The content of each physical education lesson was based on important fitness or activity components, including motor skills, aerobic fitness, strength and impact loading which are known to improve overall or specific health in children. Lessons generally included 5 minutes of warming up and cooling down each, 20 minutes of moderate to vigorous physical activity, and 15 minutes of strength training and impact loading. The physical education lessons were individualized as much as possible, so that each child was given a task possible to fulfill, based on his/her personal level. Utmost importance was given to a positive and motivating ambience during the lessons, to create any type of positive feeling and attitude towards PA.

Every INT class had one expert physical education teacher assigned. These teachers met with the "program director" (LZ) and the other main study organizers every two to four weeks to discuss the curriculum and problems that arose. Manuals were handed over to the classroom teachers regarding intervention points 1, 2 and 4 (see below) and they remained in close contact with their assigned expert physical education teachers.

Children suffering from chronic disease that prohibited the PA program, i.e. cyanotic heart disease or severe motor handicaps, were excluded. The intervention consisted of increasing PA during school, school breaks and at home. The following was applied to all 1^st ^and 5^th ^grade classes throughout one school year:

1. *Daily physical education classes*. The three already existing regular weekly physical education lessons (45 minutes each) were given by the usual classroom teachers according to a specified curriculum, with the content of the lessons determined by the expert physical education teachers. The classroom teachers had a continuous education by these experts regarding their own physical education lessons. The two additional weekly lessons (45 minutes each) were given by the expert physical education teachers. The usual classroom teachers took part in these lessons for teaching purposes.

2. Several *short activity breaks *(2–5 min each) during academic lessons were introduced every day according to a specified curriculum.

3. *PA homework *prepared by the expert physical education teachers, for improvement of motor skills, aerobic fitness, strength and jumping activities were given.

4.*Playground areas *of the schools were adapted and improved to encourage activities during school breaks, before and after school.

5. *Active commuting *to school was encouraged on the family and teachers level by flyers and posters in the schools.

6. *Families *were encouraged to be physically active through flyers which were distributed over the year.

7. The expert physical education teacher encouraged the children on two occasions to *decrease media use time *in general. One flyer was handed to the children promoting less television time and more PA.

Furthermore, minimal basic nutritional information and advice was handed out to all children and their parents (INT and CON group) in the form of three flyers. These flyers comprised information about: 1. general healthy nutrition and the nutritional pyramid, 2. appropriate calcium and Vitamin D intake for healthy bones and, 3. healthy snacks for the school breaks.

The CON group continued to follow their usual school curriculum which included three physical education lessons (45 min each) per week, given by their classroom teachers. Children and parents were blinded regarding the existence of intervention schools. The teachers, however, knew about the intervention arm. Children and parents were told that the aim of the study was the assessment of PA, fitness and health of Swiss children.

### Measurements

All measurements, i.e. at baseline, at the end of the intervention and again three years later were done within the same three weeks period for INT and CON children. Table 1 gives an overview about all measurements taken. We defined three groups for the different assessments (medical team, questionnaire team, fitness team) who worked in parallel in different schools. In each team, the same one to two main research assistants were present at every assessment, to minimize inter-observer variability. The medical team was responsible for anthropometry, cardiovascular risk assessment, blood and urine sampling and accelerometry. The questionnaire team was responsible for the distribution, help in completion and collection of all questionnaires. The fitness team performed the fitness testing. Due to the immense personnel used to perform all assessments, only a part of the researchers were blinded to group allocation. Blinded measurements included height and weight assessment, bioelectrical impedance, dual energy x-ray absorptiometry, and some of the fitness tests. Formal, standardized training of all people involved in the measurements was done in a pilot study, including two school classes with 7- to 11-year-old children three months prior to baseline assessment.

#### Measures

##### Physical activity

Physical activity was assessed by an accelerometer (MTI/CSA 7164, Actigraph, Shalimar, FL, USA) which was constantly worn around the hip over 7 days at baseline (summertime), 5–6 months into the intervention (wintertime) and at the end of the intervention both in the INT and the CON group. The 6 months measurements were done in order to be able to find variations of PA levels among seasons. The sampling epoch was set at one minute, and data were included if at least 4 full days (at least 3 weekdays and one weekend day) of measurements with a minimum of 12 hours for the weekdays and 10 hours for the weekends were measured [27]. The minimal measure time for the weekends was less, as most of the children slept 1–2 hours longer on these days. Time with over 30 min of continuous zero values was erased. Correlation coefficients between the CSA and indirect calorimetry are 0.62–0.93 and against direct observation 0.80–0.97 [28]. Overall PA was expressed as total counts per day and average counts/min. Moderate to vigorous physical activity as well as vigorous physical activity was determined based on time above different proposed, previously published cut-off levels for children [27].

##### Overall physical fitness

Overall fitness, as listed below, was assessed by a combination of the Eurofit test^1 ^[29], the Körper-Koordinationstest^2 ^[30] and the "Allgemeiner Sportmotorischer Test für Kinder"^3 ^[31]. All tests were performed in the gyms of the corresponding local schools with the same equipment. For 8 tests out of these 11 tests, the better result of two trials was used, unless mentioned otherwise. The tests number 5, 9 and 11 were maximal tests and thus only performed once. Test results were compared to published norm values for European children [32,33]. The following tests were included:

1. **Balancing backwards^2^: **this test of coordination included balancing backwards on 3 m long bars with a width of 3, 4.5 and 6 cm, respectively. The number of steps until the child's foot touched the floor was counted; 3 trials were performed for each bar width, and the sum of these 9 tests was used.

2. **Pushing the medicine ball^3^: **holding a 1 kg (and for children aged 11 years or older also a 3 kg) medicine ball against the chest and pushing it away as far as possible. The pushing distance was measured in cm.

3. **Throwing the tennis ball^3^: **throwing a tennis ball towards a 60 cm wide square target from a distance of 3 m. Hitting the inner centre of 10 cm yielded 3, the intermediate square 2 and the outer square 1 point, respectively. The total score of 10 tests was used.

4. **Jumping sidewards^2^: **jumping with both legs together on alternating sides of a strip of wood, as many times as possible, within 15 seconds. The number of jumps was counted.

5. **Sit-ups^1^: **maximum number of sit-ups achieved in 30 seconds.

6. **20 m sprint^3^: **electronic measurement of a timed (seconds) 20 m sprint, starting from a resting position, with a precision of 1/100 second.

7. **Plate tapping^1^: **the time needed for tapping 2 plates (edges were 60 cm apart) alternately, with the preferred hand, until each plate was touched 25 times. Time in seconds was measured to the nearest of 1/10 of a second.

8. **Jump and reach^3^: **the difference in height between standing (baseline) and after a maximal jump (endpoint) with arms rose on both occasions. The difference between baseline and endpoint in cm was measured.

9. **Bent-arm hang^1^: **maintaining a bent arm position with an over-grip as long as possible while hanging from a bar. The duration in sec with a precision of 1/10 of a second was measured.

10. **Sit and reach^1^: **bending the trunk and reaching forward as far as possible while sitting on the floor with stretched legs and with the feet placed against a test box. A ruler was placed on the top of the box. The difference between the feet soles and the tip of the largest finger was measured in cm.

11. **Shuttle-run-test^1^: **A 20 m shuttle run was applied to all children [34]. It is a validated test [35] which measures aerobic capacity by running forth and back for 20 m, with an initial running pace of 8.0 km/h and a progressive 0.5 km/min raise of the running speed given by a sound. The maximal performance was reached when the child did not cross the 20 m line at the moment of the beep for two consecutive 20 m distances. Numbers of "paliers" (1 palier≅1 minute) performed were counted with a precision of 0.5 paliers.

##### Anthropometry and body composition

Standing and sitting height were measured by a wall-mounted stadiometer (Seca, Basel, Switzerland, accuracy 0.2 cm) and body weight was determined using an electronic scale (Seca, Basel, Switzerland; accuracy 0.05 g). Maximal armspan was measured by extending the arms looking against a wall where a movable tape was fixed. Waist circumference was measured by a flexible tape at the natural waist (smallest circumference between the ribcage and the iliac crest) and half-way between the lower cost rim and the Spina iliaca anterior superior. Inter-observer coefficient of variation was 0.7–1.6% in the pilot study. Skinfold thickness was measured in triplicate to the nearest 0.5 mm with Harpenden calipers (HSK-BI, British Indicators, UK) calibrated to exert a pressure of 10 g/cm^2 ^to the skin. Four sites over triceps, biceps, subscapular and suprailiacal were measured based on standard procedures. The same two investigators took all measurements. Inter-observer coefficient of variation tested in a pilot study was 5.6% for biceps, 4.7% for triceps, 7.5% for subscapular and 14.6% for suprailiacal sites, respectively. The sum of the four SF was taken to calculate percent body fat (%BF) according to several validated formulas [37,38]. The calculation of %BF with this method has a prediction error of 3–5% [37,38]. Bioelectrical impedance was measured by a tetrapolar single frequency device (RJL Systems, Model 101A; Detroit, MI, USA). The unit was calibrated prior to each testing day using a 500-ohm resistor provided by the company. Measurements were taken based on standard procedures (Bioelectrical Impedance analysis in body composition measurements [39]. If the distance from the proximal to the distal electrode was less than 5 cm in small children, the proximal electrode was located more proximal until the distance of 5 cm was attained. %BF was calculated based on validated formulas [40]. The calculation of % BF with this method has a precision error of 4–5% [40]. Additionally, body composition was determined by means of bioimpedentiometric analysis at frequencies of 1, 5, 10, 50 and 100 kHz using a tetrapolar impedance plethysmograph (In Body 3.0; Biospace, Seoul, Korea) and standardized procedures. Total and regional body fat was also measured using dual-energy x-ray absorptiometry. Subjects were scanned by the use of a whole body DXA system (model QDR-4500, Hologic, Waltham, MA, USA). They laid supine on the DXA table with arms adequately separated from the trunk and were instructed to remain still throughout the scanning procedure. Body composition was assessed by the 3-compartement model, including fat mass, bone mineral content and fat-free soft tissue. DXA was found to be reliable in children [41] and sensitive for changes of fat and lean body mass [42]. For determination of abdominal fat mass a quadrilateral box was manually drawn around the L1–L4 region of interest bounded inferiorly by the horizontal line identifying L4/L5 vertebral space and superiorly by the horizontal line identifying the T12/L1 vertebral space [43].

##### Blood pressure

Blood pressure was assessed after a rest period of five minutes at the right arm based on recommendations of the American Heart Association. An automated oszillograph (Oscillomate, CAS Medical Systems, Branford, CT, USA) was used in order to reduce inter-observer variability. Blood pressure was measured five times and the mean of the three measurements with the smallest variation was taken. Systolic and diastolic blood pressures were expressed in percentiles [44]

##### Assays

Blood sampling was done early in the morning between 7.30 and 9.30 am after an overnight fast and at least 30 minutes after application of an anaesthetic cream with lidocaine and prilocaine (Emla, Astra, Switzerland). After the procedure, a breakfast was served to all children.

Bloods were drawn for measurements of glucose, insulin, lipid, highly sensitive C-reactive protein (hs-CRP), adiponectin and sex hormone binding globulin (SHBG). The blood was collected in vacutainers which were immediately put on ice. After the sampling, the blood was transported to the hospital where it was analyzed within a few hours after sampling. The rest of the blood was centrifuged, divided into 1.0 ml aliquots and stored at -70°C until batch-analyses. Insulin was measured by electro-chemiluminescence (Roche-Diagnostics, Rotkreuz, Switzerland). The reference range for insulin is 17.8–173 pmol/L (2.6–24.9 μU/ml), the intra-assay coefficient variation (CV) is 1.90% and the inter-assay CV is 2.60%. This insulin test is highly specific and has no known cross-reactions to proinsulin. Serum glucose was measured by the hexokinase method. Insulin resistance (IR) was estimated by calculating homeostasis model assessment (HOMA-IR) index (fasting serum insulin (μU/ml) × fasting plasma glucose (mmol/liter)/22.5). SHBG concentrations were measured by electrochemiluminescence immunoassays (Roche-Diagnostics, Rotkreuz, Switzerland). The intra-assay CV of SHBG concentrations are 2.7% and the inter-assay CV is 5.6%. Hs-CRP was measured automatically by a nephelometric latex immunoassay (Roche-Diagnostics, Rotkreuz, Switzerland). The functional sensitivity of hs-CRP is 0.11 mg/L, and the reference range was less than 5 mg/L. The intra-assay CV of hs-CRP is 1.34% and, the inter-assay CV 5.70% at the cut-off of the reference range. Adiponectin was measured with an ELISA (R&D Systems, Minneapolis, USA). At the lower reference range the intra-assay CV is 2.5% and the inter-assay CV 6.8%. Samples for each subject were run in the same assay. Total and high density lipoprotein (HDL) cholesterol and triglycerides were measured with a homogenous enzymatic colorimetric test, while low density lipoprotein (LDL) cholesterol will be calculated by the Friedewald's formula. Within assay and between assay CV is 0.8 and 1.7% for total cholesterol, 0.7% and 1.6% for HDL, 1.5% and 1.8% for triglycerides, respectively. Children were asked if they had accidentally eaten or if they suffered from any acute infectious illness. The respective samples of these children were removed from further analyses.

##### Bone health

Bone mineral content (BMC, g), bone area (BA, cm^2^) and bone mineral density (BMD, g/cm^2^) were determined by dual energy x-ray absorptiometry (DXA) using a Hologic QDR-4500 instrument (Waltham, MA, USA). The following skeletal sites were assessed: total body, femoral neck and trochanter, and L1–L4 vertebrae in antero-posterior view as reported previously [45]. The coefficient of variation of repeated measurements at these sites as determined in young healthy adults varies between 1–1.6% for BMD, and 0.3–3% for BMC and BA [46].

For the bone markers, a morning spot urine was collected in addition to the blood samples. From the sera, we measured procollagen type I N-terminal peptide (PINP) by antibody radioimmune assay (Orion Diagnostics, Espo, Finland) collagen type I telopeptide (CTX), N-MID-osteocalcin (OC), intact parathormon (PTH) by electro-chemiluminescence immunoassay (ECLIA) in an automated analyzer (Roche Diagnostics, Rotkreuz, Switzerland) and vitamin D (25-OH-Vit D) by RIA (25-Hydroxyvitamin D RIA Kit, Diasorin, Stillwater, MN, USA). Extraction of 25-OH-D and further hydroxylated serum metabolites were performed through acetonitril. We furthermore measured alkaline creatinine phosphatase in urine and serum by auto-analyzer (Hitachi 911, Roche Diagnostics, Rotkreuz, Switzerland), urine N-telopeptid (NTX) of type I collagen/creatinine by ELISA (Osteomark NTx, Ostex International, Seattle, WA, USA), pyridinium collagen crosslinks (pyridoline, PYD; desoxypyridoline, DPD)/creatinine in the urine by „high performance liquid chromatography" (HPLC) with a kit of BioRad Laboratories (München, Germany).

#### Questionnaires

All questionnaires were distributed by school classes in coded envelopes. The PA and psychosocial health questionnaires of the 5^th ^graders were filled out by the children themselves. To prevent bias from the parents, the psychosocial questionnaire for the 1^st ^graders was filled out at school. The rest of the questionnaires (personal and family history, quality of life, food frequency and calcium questionnaire, PA of 1^st ^graders and parents, questionnaire about school) were taken home and filled out by the parents. Questions and answers for this questionnaire were read in front of the child and the answers were filled out by the researcher. Questionnaires were sent in two batches of different sets to the parents, within one week interval to spread the load of questions. The teachers collected the questionnaires some days later. Translation of the questionnaires was not done, because only a small number of parents had insufficient knowledge of the German language, and if so, they got help from the school social workers, from the teachers or from friends.

##### Physical activity

The PA questionnaire was composed of several validated questionnaires [47,48]. In addition, we added questions about physical activities performed outdoors, the way of commuting to school, as well as questions about inactivity behavior with duration (minutes) and frequency (i.e. times per week) in front of TV or computers. The parents also got a questionnaire to evaluate the nature and extent of their own PA and their attitude towards PA of their children.

##### Personal and family history

This questionnaire included information about birth weight and pregnancy week recalled by the parents, personal history for injuries and disease (i.e. back pain, cardiovascular or bone disease in the family), weight and height of family members, as well as education and profession of the parents. Included in this questionnaire was also a sheet with the Tanner stages on which children and parents had to rate the pubertal stage of the child (only for the 11-year-old children).

##### Psychosocial health and quality of life

This questionnaire included scales of published and validated questionnaires to assess social indicators of psychosocial health which might interact with daily PA, fitness and health of school-children, such as health-related indicators of quality of life [49,50], social climate in school [51], coping strategies with stress [52], social anxiety [53], depressed mood, self-esteem [54] and substance abuse [55].

##### Food and calcium intake

Daily food intake was assessed by an abbreviated form of the CARDIA food frequency questionnaire [56], adapted to Swiss nutrition patterns (this was done based on our own experience, the experience of other pediatricians and on the experience of a preschool-based intervention program. Briefly, parents were asked to report where breakfast, dinner and evening meals were eaten, whether meals were cooked, and how frequently meals were eaten in fast food or normal restaurants. According to the CARDIA food frequency questionnaire, we also assessed the frequency of weekly servings of fruits, raw and cooked vegetables, whole grain bread, refined grains (white bread, corn flakes), of foods typical of fast foods (pizza, hamburgers, cheeseburgers, chicken nuggets, french fries, other fried food), of foods with a content high in saturated or total fat or sweets (nuts, sausages, potato chips, chocolate, ice cream, cookies, cakes, lollipops, other sweets), and of beverages (sugared carbonated drinks, fruit juices, chocolate drinks). Calcium intake was assessed by a validated questionnaire [57] adapted to the Swiss nutrition pattern.

##### Diary for school absence

All reasons for any absence from school and physical education classes was noted continuously by the classroom teachers. When the child regained school, a questionnaire about reasons for the absence from school or physical education was handed to the child and filled out by the parents. In case of a visit to a physician, these physicians were asked to fill out a form with the main complaints and the diagnosis. The sheet was returned to the study physician in a closed envelope. Teachers noted information regarding all causes of reduction in physical education of more than a day.

## Discussion

As suggested by world experts who met to define the minimum time spent in PA to secure health and to prevent unhealthy weight gain [3], the school is an ideal setting in which environmental changes to increase PA and decrease sedentary behaviour in children can be implemented. A public school-based PA intervention offers a good opportunity to work with a large group of average children irrespective of their parents' behaviour and attitudes towards PA and health, and irrespective of their socioeconomic background. This is important for many reasons. Although cross-sectional studies have emphasized the role of the family regarding PA [58] and some of the consequences of insufficient PA such as childhood obesity, approaches focusing on changing individual or family behaviours have been shown to be time-consuming and costly, and have only yielded few successes [26]. Interventions to change lifestyle habits through a change in environment, such as the school environment, are found to be more successful, especially if they take place early in life [59]. Secondly, it seems that the socioeconomically deprived children are those who experience the greatest reduction of PA and some of its consequences and are also most difficult to reach by any preventive measure that is not included in the regular school curriculum.

The increase in PA during school hours for all children and our attempt to encompass out-of school behaviour allows us to test, whether a compensatory decrease in PA outside school can be avoided, and whether outcome is different in children with different weight status, different degrees of PA and different socio-economic background. The focus on PA alone with a negligible advice regarding television viewing and nutrition, which was also given to the control group, helps us to assess the impact of changes in PA by itself in these age groups on outcome measures like childhood obesity. In contrast to interventions focusing on nutrition or media use, changing PA is less directly linked to prohibition of activities and may thus be perceived as positive rather than negative.

Our trial has some limitations. First, despite the randomization of intervention and control schools, the children, parents and classroom teachers knew the group allocation prior to baseline testing which could bias test results. This was unavoidable, as the time schedule and organization of the school year in the intervention groups had to be adapted several months before the school year started. Second, some of the investigators were not blinded to group assignment of the children because of the extensive need of manpower for testing and the financial restraints to recruit additional, blinded personnel. To assess some of the variables, we did not exclusively use validated measures. For example, we modified some of the questionnaires regarding PA or food intake to better adapt to our local situation and to broaden some of the information. Regarding PA, we will validate the questionnaires against the accelerometer measurements both before and after the intervention. Regarding the food questionnaire, we think that it is more informative to adapt them to the local use, especially as the gold standard, i.e. a 3–7 dietary recall protocol by itself has many shortcomings. PA has various effects on multiple determinants of health. We therefore defined several primary outcomes to be able to measure all important aspects related to an increased PA which we intended to induce by our intervention.

We hypothesize that our intervention will lead to an increase in PA, fitness and overall health. Should we be able to confirm these hypotheses, implementation of the PA promotion program throughout Switzerland and in other countries will help to improve health and fitness of our school children with the obvious potential of reduced direct (health care) and indirect (work absenteeism and productivity) costs later in life.

## Competing interests

The author(s) declare that they have no competing interests.

## Authors' contributions

LZ, JP, UP and SK designed the study, participated in the coordination of the study and in writing of the article. MS, MK, RG and RR conducted the study, established methods and questionnaires of the study and participated in writing the up the article. All authors provided comments on the drafts and have read and approved the final version.

## Pre-publication history

The pre-publication history for this paper can be accessed here:



## Figures and Tables

**Table 1:** Overview of measurements

**Physical activity**	**Cardiovascular risk factors**
Extent and pattern*	Body composition
Accelerometers	Blood pressure
	Glucose
**General fitness**	Insulin
Aerobic fitness	Adiponectin
Strength	Lipids
Balance	Highly sensitive CRP
Coordination	SHBG
Speed	
	**General health**
**Anthropometry**	Personal and family history*
Standing and sitting height	Nutrition*
Weight	Missed school due to injury or illness*
Armspan	Back pain*
Waist and hip circumference	
Pubertal stage*	**Psychosocial health**
	Quality of life*
**Bone health**	Self-esteem*
Calcium intake*	Social fear*
Densitometry	Stress management*
Markers of bone metabolism	School ambience*
History of bone disease*	Substance abuse*

*evaluated by questionnaire

CRP = C-reactive protein, SHBG = sex hormone binding globulin
